# *Expression of Concern:* Liu, X., Zhong, L., Li, P., & Zhao, P. MicroRNA‐100 Enhances Autophagy and Suppresses Migration and Invasion of Renal Cell Carcinoma Cells via Disruption of NOX4‐Dependent mTOR Pathway, *Clin*. *Trans. Sci*. https://ascpt.onlinelibrary.wiley.com/doi/full/10.1111/cts.12798

**DOI:** 10.1111/cts.13217

**Published:** 2022-01-17

This Expression of Concern is for the above article, published online on May 1, 2020 in Wiley Online Library, and has been published by agreement between the journal Editor‐in‐Chief, Dr. John Wagner, and John Wiley & Sons, Inc. The expression of concern has been agreed to due to concerns about the validity of the Western blot and qPCR data presented in the article, along with similarities to other CTS articles that have been retracted or for which an expression of concern has been published. In accordance with Committee on Publication Ethics (COPE) guidelines, the journal has asked all authors and their institutions for raw data that may answer some of the questions raised. Upon multiple requests, the authors have submitted raw data for the editorial team’s interpretation. The editorial team found the provided data inconclusive. As a result, the journal has decided to issue an Expression of Concern to readers. Readers should interpret the information presented in the article with due caution.

